# Eclectic Department

**Published:** 1851-11

**Authors:** 


The "Dublin Journal" gives the following diagnosis of Asthma and Œdema of the Glottis, as contradistinguished from each other. — *N. Y. Med. Gazette.*

DIAGNOSIS BETWEEN SPASMODIC ASTHMA AND SUBMUCOUS LARYNGITIS OR ŒDEMA OF THE GLOTTIS.

A. *Symptoms in Common.* — The patient is seized with a fit of suffocation, which soon attains its highest degree. The countenance becomes congested, the eyes starting from the orbits, and at the same time the extremities become cold; in short, presenting the appearance of asthma in its severest form.

B. *Distinguishing Symptoms.*

| In Œdema of the Glottis. | In Asthma. |
|---|---|
| 1. Inspiration produces no painful sensation in the chest, but only in the larynx, to which the patient refers all his difficulty in breathing. | 1. The impediment to inspiration is proved to be not so much in the larynx as in the chest. |
| 2. Every effort to draw in the breath is accompanied by a wheezing sound. | 2. Inspiration takes place without any peculiar sound in the larynx. |
| 3. Expiration goes on uninterruptedly. | 3. Expiration and inspiration are equally difficult. |
| 4. The patient thinks there is some large foreign body in his larynx, which he would gladly get rid of. | 4. No sensation as of a foreign body in the larynx. |
| 5. The suffocative attack lasts only from two to four minutes. | 5. The asthmatic attack lasts several hours. |
| 6. It returns in from five to ten minutes. | 6. It returns at the utmost in about every twenty-four hours. |
| 7. The cough is dry and of a croupy sound. | 7. The cough moist and with the usual sound. |

